# Dissociable roles for lateral orbitofrontal cortex and lateral prefrontal cortex during preference driven reversal learning

**DOI:** 10.1016/j.neuroimage.2011.10.072

**Published:** 2012-02-15

**Authors:** Adam Hampshire, Amir M. Chaudhry, Adrian M. Owen, Angela C. Roberts

**Affiliations:** a Department of Physiology, Development and Neuroscience, University of Cambridge, Downing Street, Cambridge, CB2 3DY, UK; b Medical Research Council Cognition and Brain Sciences Unit, Chaucer Road, Cambridge, UK; c Behavioural and Clinical Neuroscience Institute, Downing Street, University of Cambridge, CB2 3EB, UK; d Centre for Brain and Mind, University of Western Ontario, London, Ontario, Canada

**Keywords:** Reversal learning, fMRI, Executive function, Attention, Frontal lobe

## Abstract

One of the archetypal task manipulations known to depend on frontal-lobe function is reversal learning, where a dominant response must be overridden due to changes in the contingencies relating stimuli, responses, and environmental feedback. Previous studies have indicated that the lateral prefrontal cortex (LPFC), the lateral orbitofrontal cortex (LOFC), the anterior cingulate cortex (ACC), and the caudate nucleus (CN) all contribute to reversal learning. However, the exact contributions that they make during this cognitively complex task remain poorly defined. Here, using functional magnetic resonance imaging, we examine which of the cognitive processes that contribute to the performance of a reversal best predicts the pattern of activation within distinct sub-regions of the frontal lobes. We demonstrate that during reversal learning the LOFC is particularly sensitive to the implementation of the reversal, whereas the LPFC is recruited more generally during attentional control. By contrast, the ACC and CN respond when new searches are initiated regardless of whether the previous response is available, whilst medial orbitofrontal cortex (MOFC) activity is correlated with the positive affect of feedback. These results accord well with the hypothesis that distinct components of adaptable behaviour are supported by anatomically distinct components of the executive system.

## Highlights

► We model fMRI data at distinct stages of a reward driven reversal learning task. ► Lateral orbitofrontal cortex responds particularly strongly at the point of reversal. ► Lateral prefrontal cortex shows a similar response during other switches. ► Medial orbitofrontal cortex activity correlates with the rewarding value of feedback. ► Anterior cingulate cortex and caudate respond whenever new searches are initiated.

## Introduction

The ability to alter behaviour according to changes in the environment is important for the survival of any organism. One of the classic measures of behavioural flexibility is reversal learning, where a dominant response is overridden in favour of a weaker competing alternative due to a change in feedback contingencies. Cortically, it is now well established that damage to the ventral prefrontal cortex leads to impairments in visual discrimination reversal learning in humans (Fellows and Farah, 2003; Hornak et al., 2004), monkeys (Dias et al., 1996; Iversen and Mishkin, 1970; Izquierdo et al., 2004) and rats (Chudasama and Robbins, 2003; Schoenbaum et al., 2002). This is mirrored by functional neuroimaging studies in humans that show a change in blood flow in a network of brain regions at the point in time when subjects first switch their responding following a reversal of the reward contingencies. This network includes the lateral orbitofrontal cortices (LOFC) (Chamberlain et al., 2008; Ghahremani et al., 2010; Hampshire and Owen, 2006; Hampshire et al., 2008a; O'Doherty et al., 2001) and the lateral prefrontal cortex (LPFC) (Budhani et al., 2007; Cools et al., 2002; Ghahremani et al., 2010). Indeed, multiple regions within the inferior frontal cortex may contribute to reversal learning. Thus, ablations of either areas 11, 13 and 14 (medial orbitofrontal cortex MOFC), or area 12 (including LOFC and LPFC) in rhesus macaques, disrupt reversal learning (Iversen and Mishkin, 1970; Izquierdo et al., 2004); albeit with a different pattern of errors (for review see Clarke and Roberts, 2011). In support of this hypothesis, we have shown that the effects on reversal learning of excitotoxic lesions of area 12 (LPFC and LOFC) and areas 11 and anterior 13 (anteromedial OFC) in the new world monkey, the common marmoset, can be differentiated according to the level of prior reversal learning experience (Rygula et al., 2010). However, the separable contributions of LOFC and LPFC have not been determined. In addition, the precise contributions of other regions outside of the ventral prefrontal cortex that have also been implicated in visual discrimination reversal learning, in particular, the anterior cingulate cortex (ACC) and the caudate nucleus (CN) (Clarke et al., 2008; Cools et al., 2002; Rogers et al., 2000) remain unclear.

The reversal learning process, whilst providing a useful gauge of behavioural flexibility, is cognitively heterogeneous, being composed of multiple sub-processes. For example, successful reversal performance requires a participant to process task feedback and to instigate searching for an optimal behaviour by overriding a dominant response and reorienting the focus of attention. Thus, in order to better specify the neural basis of reversal learning, the present study sought to determine how anatomically distinct components of the executive system responded during the cognitively separable stages of the reversal learning process. In particular, we wanted to determine whether activations in both the LPFC and LOFC were specifically related to feedback driven learning in the presence of contingency change or not. Accordingly, participants undertook a series of visual discriminations and reversals in which they had to select one of a set of abstract patterns, subsequent to which they received feedback in the form of either an attractive or an unattractive female face. The reward contingencies were probabilistic and reversed unpredictably after a variable number of consecutive correct responses. This design enabled us to examine whether the BOLD responses were related to i) the processing of negative feedback in general, ii) the initiation of a new search iii) switching response from one object to another in the presence of a prepotent response (reversal) or iv) switching response from one object to another, in the absence of a prepotent response (acquisition). Such a comparison has not been possible in the majority of previous imaging studies of reversal learning, because these studies investigated either the serial reversal learning of a single discrimination (Cools et al., 2002; O'Doherty et al., 2001) — thus lacking a suitable control for (ii) and (iv), used an absolute reward contingency (Hampshire and Owen, 2006) preventing the independent analysis of (i) and (iii) or presented stimuli concurrently (Ghahremani et al., 2010) not allowing for comparison of (iii) and (iv).

## Methods

### Participants

Nineteen healthy right handed, male volunteers with no history of psychiatric intervention or neurological illness participated in this study (mean age 29 ± 6 youngest 19, oldest 40). All participants gave written consent prior to taking part and the study was approved by the Hertfordshire Local Research Ethics Committee.

### Experimental design

**Fig. 1:**
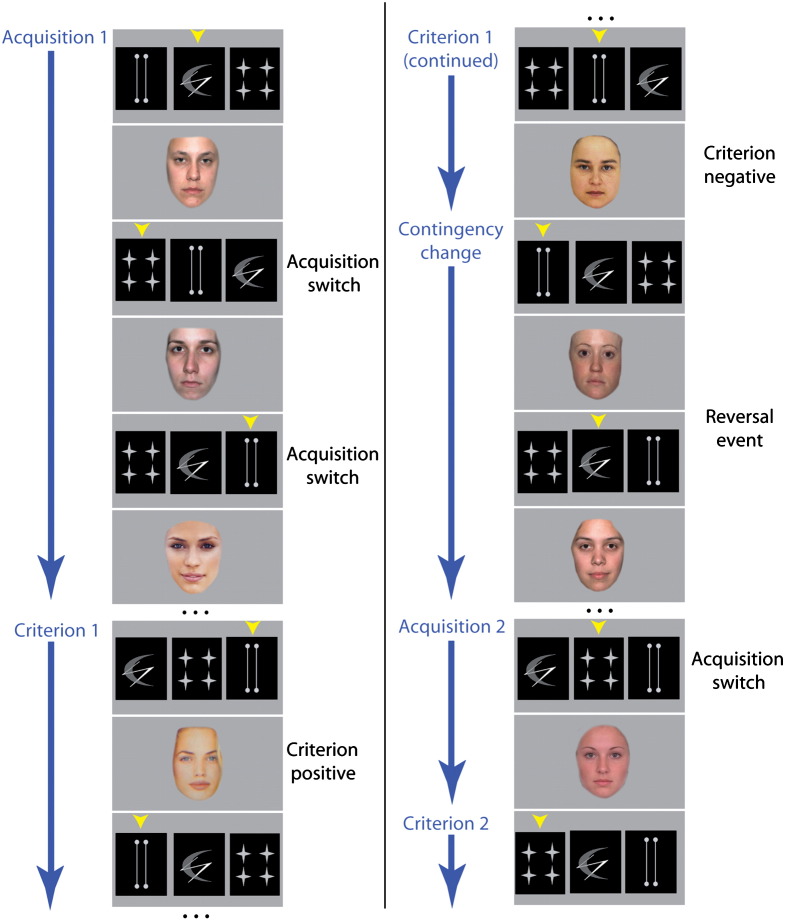
Fig. 1 illustrates the task design. Participants selected one of three abstract patterns that were positioned horizontally across the screen. Subsequently, feedback was presented in the centre of the screen in the form of either an attractive or an unattractive female face. At any given stage of the task there was a rule defining the contingency between pattern selection and feedback such that selecting one of the patterns led to an attractive face five out of every six responses, whereas selecting one of the other patterns always led to the reception of an unattractive face. Each block was divided into five distinct phases. In the first phase, (Acquisition 1) the participant explored the rule relating the selection of the different available patterns to the reception of attractive or unattractive faces. During this phase they made a number of ‘acquisition switches’, switching from one stimulus to another across trials, before they settled on consistently selecting the same pattern and entered Criterion 1 Phase. This phase lasted until participants had made 5 or 6 consecutive responses to the same pattern, during which they received the positive ‘attractive face’ on most occasions, ‘criterion positive’ but at least once, received an unattractive face, ‘criterion negative’. Then, the contingencies relating patterns to feedback were changed — (Contingency change) and upon realising that this was the case, the participants performed a reversal by switching their responses away from the previously rewarded pattern and selecting an alternative pattern, ‘reversal event’. The participant then entered the fourth stage of the block (Acquisition 2) in which they explored the new rule relating pattern selection to feedback, subsequent to which they entered the final stage (Criterion 2) in which they settled on consistently selecting the same pattern. When the criterion of 5 or 6 correct responses was met a new block began in which the participants were presented with a completely novel stimulus set. Black dots represent additional trials within a phase that are not depicted in the figure.

On an individual trial, a stimulus set containing three abstract patterns was displayed (Fig. 1) and the participant responded by selecting one of the patterns using the first three fingers of their right hand on a button box. At the point of response the patterns were removed from the screen and positive or negative feedback was received. This was in the form of a picture of either an attractive or an unattractive female face presented in the centre of the screen for 2 s. Overall, males prefer to view attractive female faces (Aharon et al., 2001) and in the present study they preferentially selected the patterns that led to presentation of faces from the attractive category. For any individual feedback event the specific face that was presented varied, having been selected from a pool of either 125 attractive faces or 51 unattractive faces (see below). After a short inter-stimulus interval, a new trial began, and the patterns reappeared on the screen, pseudo-randomly reshuffled into the three horizontal positions with the constraint that the same pattern could not appear in the same location 3 times in a row, nor could the order of patterns be the same on any two consecutive trials. The trials were arranged in blocks so that during any given phase of the task there was a rule defining which of the patterns from the stimulus set was ‘optimal’, being the most likely to lead to the reception of positive feedback. The feedback contingencies were set so that selection of the optimal pattern led to positive feedback five times out of every six and negative feedback one time out of every six. By contrast, selecting one of the other patterns always led to negative feedback. Participants were not informed of these contingencies and were simply told to respond however they wanted to. Once the same pattern had been selected five or six times in succession, including at least one misleading feedback trial, it was deemed that a routine response had been established. These responses had to be consecutive with no response to an alternative pattern in between. Subsequently, the rule determining which pattern was optimal, changed. Hence, continued selection of the previously chosen pattern now led to the presentation of an unattractive face, whereas selection of one of the patterns that had previously led to presentation of an unattractive face now led to presentation of an attractive face most of the time. After this change in the rule, the reward contingencies remained unchanged until a criterion of 5 or 6 consecutive responses to the newly optimal pattern was reached. At this point a completely new set of patterns was presented, marking the beginning of a new block of trials.

Each block, therefore, consisted of five distinct phases of behaviour. In the first phase ‘acquisition 1’ the participant explored the outcomes relating to selection of the different patterns. In the second phase ‘criterion 1’, the participant settled on selecting repeatedly the same pattern that they deemed most likely to lead to positive feedback. In the third phase ‘contingency change’ the rule defining which pattern was most likely to lead to positive feedback changed, and the participant determined, on the basis of the negative feedback, that a change in behaviour would lead to a more rewarding outcome. This third phase culminated in a reversal event in which the participant inhibited the dominant routine response to the previously rewarded pattern and started selecting one of the other patterns. The participant then entered the fourth phase ‘acquisition 2’ in which they explored the new outcomes when selecting the patterns, subsequent to which they entered the fifth and final ‘criterion 2’ phase of consistently responding to the pattern that led to the most rewarding outcome. Only those blocks in which all five phases were successfully completed were included in the event related fMRI analysis.

In half the blocks, although three patterns were presented on the screen, only two were available for selection since one of them was considered inactive, denoted by being covered in small crosses. Although any responses to the inactive pattern were recorded they did not lead to any change of the display. These blocks alternated such that after participants had completed a block of trials using 3 available patterns, they would begin a block with only 2 patterns available. This manipulation was intended to examine differences in neural response associated with the degree of choice available to the participant at the point of reversal. However, as no significant results were associated with this manipulation it will not be discussed further here.

### Face stimuli

The attractive and unattractive face stimuli were presented as colour images with direct eye gazes. To ensure similarity in emotional facial expression only faces with neutral to mild smiles were included in the stimulus set. Faces were scanned from print media, obtained from other researchers (O'Doherty et al., 2003) or selected from databases available online (Computer Vision Laboratory Face Database, University of Ljubljana (Solina et al., 2003)). Images were cropped to have little or no hair and were placed on a grey background. In total 214 images were prepared and these were presented to 13 males who met the criteria for scanning but who did not take part in the experiment. These males rated the faces on a visual analogue scale (VAS). The faces were presented individually in a randomised order on a computer screen above the VAS, which was anchored on the left as ‘Unattractive’ and the right as ‘Attractive’. All ratings were normalised to the length of the scale and faces subsequently used in the scanning task were those with a mean rating above 0.6 (125 images), defined as attractive, and those with a mean rating below 0.5 (51 images), defined as unattractive.

### Instructions

Participants were presented with instructions, both verbally and in written form, immediately before commencement of the training session. Care was taken to ensure that participants were not explicitly made aware of the attractiveness of the faces they viewed.

‘You will be presented with three patterns on the screen. Selecting one of the patterns will result in a picture being displayed on the screen and the type of picture will depend on the pattern you have chosen. There are no right or wrong answers in this task. Whenever a new set of patterns appears, you are asked to try them all and after that, you are free to choose which type of picture you wish to view. On some trials, you may notice that one of the patterns is covered in crosses. This means that the particular pattern is not active and trying to select it will not result in viewing a picture. In this case, please make your choices from the remaining two patterns.’

In addition, participants were verbally reminded: ‘When you see a new set of patterns, please select each available pattern at least once. This is to make sure you have sampled all of the available patterns. After this you may continue selecting patterns according to your own preferences. Please pay attention to the images in case things change as you go along’.

### Data acquisition

Prior to entering the scanner, all participants underwent a short training session (~ 5–10 min) to ensure that they understood and were capable of performing the task. This training session involved performance of at least two complete reversals on a laptop computer outside the scanner. All participants underwent the same training task and the patterns and faces seen during this session were not used in the scanning tasks. Once the participants had completed the scanning task, they were asked to rate all the faces on a computerised VAS (as described above).

19 participants completed the task, each of whom undertook two 18 minute sessions of scanning acquisition in a 3T Bruker Medspec s300 scanner at the Wolfson Brain Imaging Centre, Cambridge, UK. Two data sets were lost due to technical problems, leaving 17 full data sets in the fMRI analysis. A total of 1005 gradient echo T2-weighted EPI images depicting blood-oxygenation level dependent (BOLD) contrast were collected for each of two sessions and the first 18 volumes from each session were discarded to avoid T1-equilibriation effects. We collected 21 slices per volume with a slice thickness of 4 mm, inter-slice gap of 1 mm and a TR of 1.1 s. Resolution within each slice was 3.125 mm × 3.125 mm. Slices were angled away from the orbits to avoid signal dropout in the OFC due to magnetic susceptibility inhomogeneity. A T1-weighted structural MRI image was also collected for anatomical data.

Data were processed using Statistical Parametric Mapping 5 (SPM5 — Wellcome Department of Imaging Neuroscience, London). Preprocessing consisted of slice time correction, reorientation, correcting for participant motion, geometric un-distortion using phase maps (Cusack et al., 2003), spatial normalisation to the standard Montreal Neurological Institute EPI template, and smoothing with an 8 mm Full-Width Half-Maximum Gaussian kernel.

### fMRI analyses

11 regressors were entered into the individual participant's fixed effects analyses in SPM 5. The regressors were formed by convolving onsets and durations of the events of interest with the canonical haemodynamic response function. Two of the regressors were defined during the acquisition phases when the participants were exploring the reward contingencies associated with responding to the different patterns. These regressors were defined according to whether the participant selected the same pattern as on the previous response (acquisition stay) or switched to selecting a different pattern after receiving negative feedback (acquisition switch). A further three regressors were defined during the criterion phases and the contingency change phase, where the participants were repeatedly responding to the pattern that they had identified as being the most likely to lead to positive feedback. These were defined as repetitions during criterion on the basis of positive feedback (an attractive face — criterion positive), repetitions during criterion after the reception of probabilistic negative feedback (criterion negative), and repetitions in the contingency change phase after receiving negative feedback (contingency negative). Two regressors were included representing the first response of a new search after criterion. The first of these was the reversal, representing the stage in time at which the participant first selected a different pattern to the one consistently chosen during the criterion 1. The second was the first time that the new stimulus set was presented after criterion 2 and the participant selected a new stimulus from that set for the first time (stimulus-set change). These regressors were defined from the visual onset of the patterns until the time at which the participant responded. In order to identify those brain regions in which the BOLD response correlated with the perceived attractiveness of the faces, another four regressors were formed from the onsets and durations of the feedback events. The first of these was modulated according to the VAS ratings for each face in a linear manner, whilst the other three were modulated by the second, third and fourth order non-linear polynomials of the VAS scores to ensure that any non-linear correlations between reported score and BOLD signal change were accounted for.

Data for these events were extracted from anatomically defined regions of interest (ROIs) using the MarSBaR ROI toolbox (Brett et al., 2002), which takes the average beta value from all voxels within the ROI. ROIs used in the group level analyses were taken from the standard MNI templates included with the MarsBaR ROI toolbox (Tzourio-Mazoyer et al., 2002) and focused on brain regions that had previously been implicated in reversal learning. LPFC ROIs included the inferior triangularis and the inferior operculum bilaterally, whilst LOFC ROIs included the inferior orbitalis and the mid orbitalis bilaterally. The anterior cingulate cortex (ACC), the caudate body with nucleus accumbens, and the MOFC (defined from the medial orbitalis) were also included in the analysis. Extracted data were examined at the group level in a series of contrasts that differentiated between ROIs that were particularly active at reversal and ROIs that were active more generally during switching or the reception of negative feedback. ROIs that were sensitive to the absolute reward value of the stimuli were identified by examining the mean extracted beta weights from the same ROIs for the linear and non-linear VAS regressors. In all cases, results from the ROI analyses were supplemented with whole brain analyses using FDR correction at p < 0.05 FDR corrected for all voxels within the brain.

## Results

### Behavioural assessment and analysis

After the neuroimaging procedure, participants were asked to rate all the faces contained on the database using a computer based visual analogue scale (VAS). Subjective ratings of attractiveness showed a significant difference between attractive and unattractive faces (t = 20, P < 0.001) indicating that assignment of these categories had been successful.

Behavioural data were collected from those blocks in which the participant successfully completed all five phases of the block including the reversal. On average, participants made 4.8 ± 1.9 (mean & standard deviation) responses before settling on a routine response and entering the first criterion phase. In the reversal phase, participants received an average of 2.79 ± 1.2 negative feedback events before performing a reversal and entering the second acquisition phase. The average number of reversals included in the analysis per participant was 18.05 ± 2.19. Examination of trials to criterion using a 2 ∗ 2 repeated measures analysis of variance (ANOVA), in which the conditions were acquisition session (1 or 2) and number of patterns available (2 or 3), revealed that there were no significant main effects of session (F(1,16) = 1.98, p > 0.05) or available patterns (F(1,16) = 1.15, p > 0.05), nor any interaction between these two factors (F < 1).

**Fig. 2:**
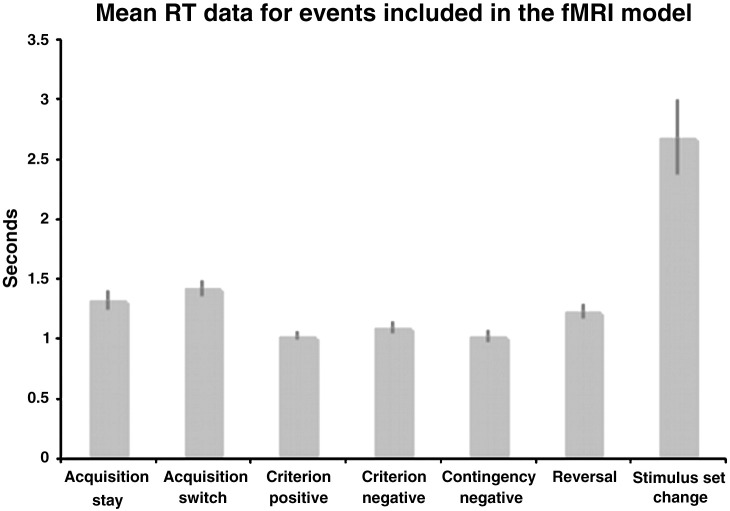
Fig. 2 illustrates the mean RT data for the different behavioural responses. Error bars represent the standard error of the mean. RTs were longer at stimulus-set change compared with reversal, contingency, and acquisition switch, presumably due to the participant taking in the new patterns. Acquisition switch RTs were significantly longer than those for contingency negative and reversal, whilst reversal RTs were significantly longer than contingency negative.

Mean response time (RT) data were calculated for the 7 types of response included in the first level models (Fig. 2). A repeated measures ANOVA carried out between the events that were compared in the group level fMRI analysis showed a significant effect of response type (F(3,48) = 27.58 p < 0.001). Pair-wise tests revealed that RTs were significantly longer at stimulus-set change compared with reversal (t = 5.2 p < 0.001), contingency negative (t = 5.7 p < 0.001), and acquisition switch (t = 4.0 p < 0.001), presumably due to the participant taking in the new patterns. Acquisition switch RTs were significantly longer than those for contingency negative (t = 5.9 p < 0.001) and reversal (t = 2.8 p = 0.013), whilst reversal RTs were significantly longer than contingency negative (t = 5.0 p < 0.001).

### Neuroimaging results

#### Contrasting regional activity at reversal to other switch conditions and to non-switches following probabilistic negative feedback

**Fig. 3:**
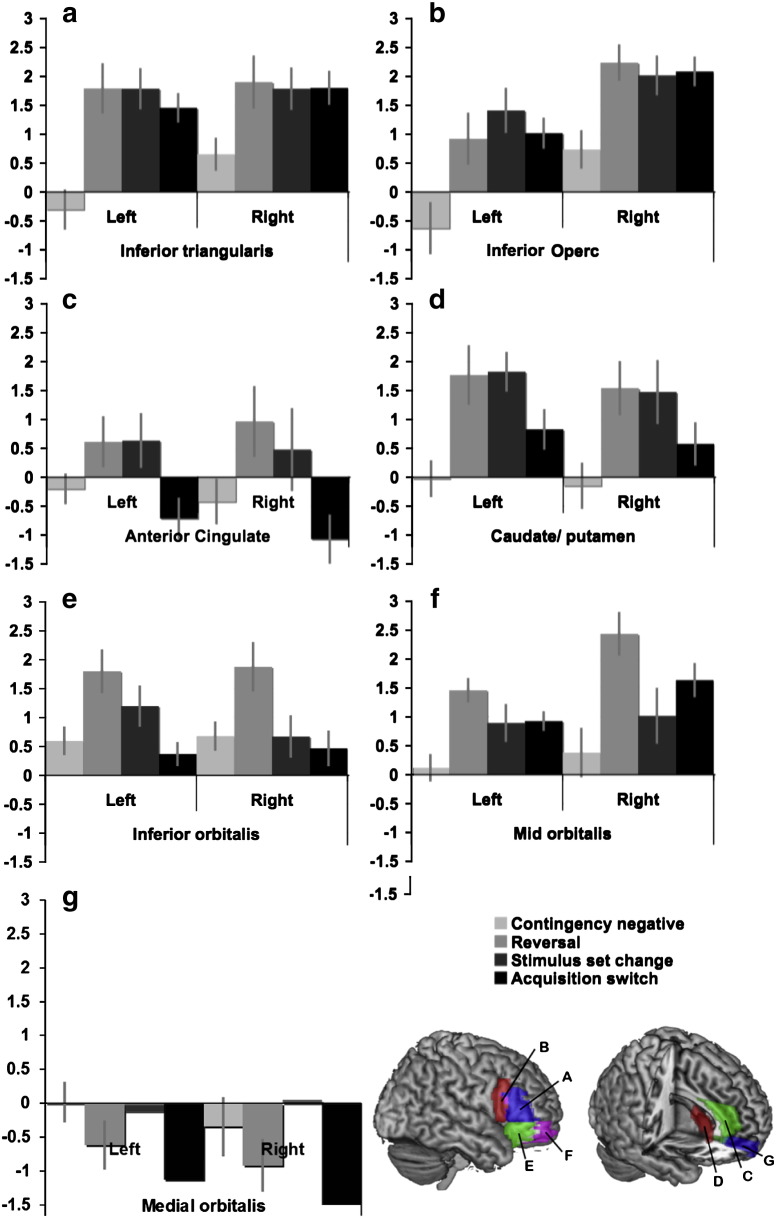
Fig. 3 illustrates the mean beta weights extracted from the ROIs for the four conditions compared in the group level fMRI analysis. ROIs showed significantly different patterns of response during different stages of the reversal learning process. LPFC ROIs consisting of the inferior operculum and inferior triangularis showed similar increases in the BOLD signal during all three switch events relative to negative feedback events that did not lead to a switch in the reversal phase. The LOFC ROIs, consisting of the inferior orbitalis and mid orbitalis, responded particularly strongly at the point of reversal, but also to a lesser extent during other switches. Both the anterior cingulate and the caudate/putamen responded strongly at reversal and stimulus-set change when new searches were initiated. The medial orbitalis did not respond positively during any of the switch conditions.

**Fig. 4:**
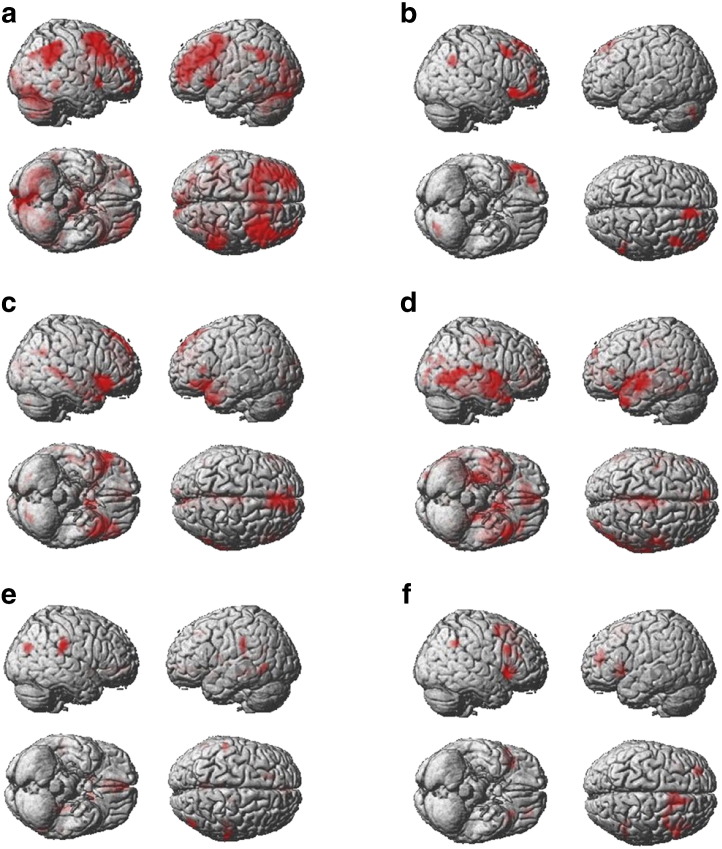
Fig. 4 reports results from the supplementary whole brain analyses with whole brain correction at FDR < 0.05. a — activation at the point of reversal relative to contingency negative events that did not lead to a reversal. b — activation at reversal relative to stimulus-set change. c — activation at reversal relative to acquisition switches. d — stimulus-set change minus acquisition switch. e — positive correlation with the linear VAS regressor. f — negative correlation with the linear VAS regressor.

We predicted that areas involved in reversal implementation in particular would be more activated at the point of reversal when compared with the non-switch events that followed negative feedback and the other switch events that did not involve overriding a routine response. Consequently, group level analyses focused on activations associated with the non-switch events that preceded a reversal (contingency negative), the reversal, responses at stimulus-set change, and acquisition switches (Fig. 3 & Table 2). First, ROIs that were recruited at the point of reversal were identified by contrasting extracted data for the reversal events with the contingency negative events that came just prior to the reversal. In both types of event, a routine response had been developed as criterion had been reached and negative feedback had been received to indicate that contingencies had changed. However, in the reversal condition the participant changed their selection for the first time, whereas in the contingency negative events the routine response was repeated. Therefore, this contrast controls for activation related to the processing of negative feedback per se. The majority of the ROIs showed significant activation at the point of reversal, with the exception of the ACC ROIs, which showed sub-threshold trends in the same direction, the right inferior orbitalis which showed an effect at just below the corrected threshold, and the Medial OFC, which showed a trend in the opposite direction (Table 1a). Examination of the same contrast using whole brain analysis with an FDR correction at p < 0.05 confirmed this result (Table 2a & Fig. 4a). More specifically, a network of frontoparietal brain regions was activated at the point of reversal, including both the lateral and orbital surfaces of the inferior frontal cortices bilaterally, the anterior cingulate cortex/pre-supplementary motor area, and the striatum. Additional areas of activation were evident in the inferior parietal cortex, the frontopolar cortex, the middle frontal gyrus, and the occipital gyrus bilaterally.

Brain regions that were particularly active during the performance of a reversal compared with other switch events were identified using two further contrasts. ROI data were first contrasted for the reversal minus the stimulus-set change events. In both cases, a routine response had first been developed, criterion had been reached, and a new search subsequently initiated. However, at stimulus-set change the routine response did not have to be overridden, as the previously selected pattern was no longer available. The inferior orbitalis and the mid orbitalis, i.e. LOFC, in the right hemisphere were significantly activated for this contrast (Table 1b). The corresponding left ROIs followed the same trend but did not stand up to correction for multiple comparisons. By contrast, the LPFC ROIs, showed equivalent activation during both types of switch event. The results from the whole brain analysis, FDR corrected at p < 0.5, supported these findings with a swathe of activation in the right LOFC including the most posterior extent of the inferior frontal gyrus extending into the anterior insula and the frontopolar portion of the middle frontal gyrus (Table 2b & Fig. 4b). More focal activation was evident in the left LOFC. Activation was also evident in the right posterior middle frontal gyrus, the superior frontal gyrus, and the right inferior parietal cortex. Unlike the reversal minus contingency negative contrast, little activation was evident in more lateral and dorsal frontal-lobe regions.

ROI data were then contrasted for the reversal minus the acquisition switch events. In both cases, the participant switched away from a previously selected pattern that was still available after receiving negative feedback, however, in the reversal, the pattern that was switched away from was more habitual, having been repeatedly selected throughout the criterion phase. Significant activation was evident selectively within the inferior orbitalis ROIs. Notably, whilst the mid orbitalis ROIs followed the same trend this effect did not stand up to correction for multiple comparisons (Table 1c). By contrast, the LPFC ROIs were activated to a similar extent in both types of switch. Interestingly, the anterior cingulate ROIs were also active for this contrast with the caudate ROIs following a similar sub-threshold trend. Whole brain analysis confirmed these results, with bilateral activation in the posterior extent of the LOFC, spreading from the most posterior extent of the IFG, through the insula, and including regions within the striatum (Table 2c & Fig. 4c). It was notable that activation within the anterior LOFC was more focal than for the reversal-set change contrast and did not extend to the frontal pole. Activation was also evident within the ACC/preSMA, and within the right middle temporal gyrus.

Intriguingly, these results suggest that the ACC and CN may be more active at both stimulus-set change and reversal when compared with acquisition switches (Fig. 3) — an unexpected finding. Stimulus-set change and acquisition switch events were therefore contrasted directly. The results demonstrated this to be the case, with heightened activation within the ACC and CN ROIs (Table 1d). Whole brain analysis showed activation within the striatum, the ACC/MOFC, the medial temporal lobe, and the middle temporal gyrus bilaterally (Fig. 4d).

#### Contrasting event-related BOLD activations to switch conditions across ROIs

In order to confirm the statistical significance of the difference in ROI activation across the four conditions, extracted data were compared using repeated measures ANOVA with factors of ROI (6 excluding MOFC), Hemisphere (left, right) and Switch Condition (contingency negative, reversal, stimulus-set change, acquisition switch). There was a large main effect of ROI (F_5,80_ = 26.2 p < 0.001), a sub-threshold main effect of hemisphere (F_1,16_ = 4.2 p = 0.056), and a significant main effect of condition (F_3,48_ = 4.6 p = 0.007). Notably, there was a large significant interaction between condition and ROI (F_15,240_ = 7.5 p < 0.001), supporting the view that different ROIs were sensitive to different task demands. There was also a significant ROI * hemisphere interaction (F_5,80_ = 16.9 p < 0.001) highlighting the right lateralisation observed in the whole brain analyses. There were no other significant interactions. To examine the question of whether LPFC and LOFC ROIs in particular were differentially sensitive to condition, data were averaged across ROI and hemisphere for the LPFC and the LOFC and then compared using a repeated measures ANOVA with factors of ROI (LPFC vs. LOFC) and switch condition. There was a significant main effect of condition (F_(3,48)_ = 8.5 p < 0.001) and importantly, a significant interaction between ROI and condition (F_(3,48)_ = 11.6 p < 0.001). Notably, there was no main effect of ROI suggesting that these results were not driven by differences in the overall mean signal intensity.

The question of whether anterior and posterior LOFC ROIs showed significantly different activation profiles across the 4 switch conditions was examined in an additional ANOVA in which the factors were switch condition (4) ROI (2) and hemisphere (2). The results showed a significant main effect of condition (F_(3,48)_ = 6.643 p = 0.001) and importantly, an interaction between ROI and switching condition (F_(3,48)_ = 13.85 p = 0.006). This interaction was driven by greater activation in the mid OFC ROI (anterior) relative to the inferior OFC ROI (posterior) in the acquisition switch condition (left t = 3.253 p = 0.005, right t = 3.036 p = 0.008).

#### Identifying brain regions in which the BOLD response correlates with absolute reward value

Sensitivity to the absolute reward value of the feedback images was examined using a series of t-tests on the mean ROI beta weights from the first, second, third and fourth order VAS regressors. The results revealed a large positive relationship between BOLD signal change and the linear VAS regressor within the MOFC ROI (Table 1e). Whole brain analysis confirmed the ROI results, with a large cluster of activation spreading from the MOFC along the medial frontal wall to the anterior cingulate (Table 2e & Fig. 4e). The right inferior triangularis and right inferior operculum showed negative correlations with the linear VAS regressor (Table 2f & Fig. 4f). There were no significant correlations between the non-linear VAS regressors and the BOLD response in any of the ROIs.

## Discussion

The use of a reversal learning paradigm in which the feedback was affective, the reward contingency probabilistic, and which employed response switching controls, allowed us to examine different stages of the reversal learning process within the same task design. In accordance with previous findings, our results implicate a network of frontal lobe and striatal brain regions in the reversal learning task. Our results also demonstrate that, whilst much of the prefrontal cortex and striatum co-activate at the point of reversal, anatomically distinct components of this network can be dissociated according to their levels of activation at other stages of the reversal learning task. These findings accord well with a model of prefrontal function in which anatomically distinct frontal lobe regions tend to support different executive demands.

### Functionally dissociating the LOFC and the LPFC

The most important result from the current study is the difference in the profile of the BOLD response in sub-regions of the inferior-frontal cortex. Whilst LPFC and LOFC ROIs responded strongly at the point of reversal, the response within the LPFC was equivalent during other types of attentional switch. By contrast, whilst the LOFC showed a significant response during all attentional switches, the response was particularly strong at the point of reversal, even when contrasted directly with switches that occurred in the acquisition phase of the task. Thus, whilst the LOFC is *differentially* activated by an attentional switch following contingency reversal, the LPFC is not. This result supports previous studies that have reported a functional dissociation between the LPFC and the LOFC during reversal leaning (Dias et al., 1996, 1997; Greening et al., 2011; Hampshire and Owen, 2006; Mitchell and Bryan, 2010; Rygula et al., 2010). Furthermore, the fact that the LPFC is recruited in a more consistent manner during all switching events, accords well with findings from our previous study (Hampshire and Owen, 2006), in which more dorsal regions of the lateral prefrontal cortex, including the inferior triangularis, showed generally heightened activation when an optimal response was being sought. By contrast, activation within the LOFC ROIs could not be attributed to response switching in general as the BOLD response was particularly strong at the point of reversal. Similarly, whilst previous hypotheses have highlighted the differential activation of the LOFC to negative as opposed to positive affective stimuli (for review see (Kringelbach, 2005)), in the current study there was little difference between non-switch events that followed positive or negative feedback prior to the reversal. This pattern of results reinforces the view that the LOFC is not specifically involved in processing negative feedback, but rather, is particularly involved when that feedback leads to a change in behaviour. This observation is also in close concordance with the findings of Liu et al. (2007), who observed that during gambling, the anterior LOFC was not recruited when negative outcomes arose as a result of betting, but later, when the urge to place a bet was overridden on the basis of that feedback.

### Is there functional specialisation within the LOFC?

These findings highlight a prominent role for the LOFC at the point of reversal. However, the question remains, of what the LOFC is doing at the process level during a reversal? One possibility is that the LOFC is involved in affecting change at the point of reversal by overriding the previously rewarded and routine response (Elliott et al., 2000; Schoenbaum et al., 2002). An alternative is that the LOFC is involved in re-evaluating current expectations about the reinforcer contingencies (Kringelbach, 2005; O'Doherty et al., 2001, 2004). Indeed, this latter hypothesis of a role in contingency evaluation is supported by both lesion studies in rhesus monkeys (Noonan et al., 2010) and human imaging studies (Windmann et al., 2006). It is also notable that OFC lesions in man have been associated with a deficiency in the ‘extinction process’ that is — the relearning of contingencies (Nahum et al., 2009). Such lesions are associated with a tendency to keep behaving according to one set of beliefs even when it is clear from environmental feedback that those predictions no longer reflect the reality of the situation (Schnider, 2003). Similarly, patients with obsessive–compulsive disorder (OCD) have both structural and functional abnormalities within the LOFC (Chamberlain et al., 2008) and are characterised by behavioural rigidity when plans must be adapted. Taken together, this evidence would appear to lend weight to the contingency re-evaluation account of LOFC function. However, it is important to note that these two interpretations are not mutually exclusive, with one relating to the nature of information processing within the LOFC, and the other relating to how LOFC output may affect processing within other brain regions. Furthermore, it seems likely that the LOFC itself is a cognitively heterogeneous structure (Kringelbach, 2005). For example, within the current data set, it is notable that the anterior and posterior LOFC, whilst both responding most strongly at the point of reversal, showed significantly different response profiles during other types of switch. More specifically, the mid orbitalis ROIs showed a greater response during acquisition switches than the more posterior inferior orbitalis ROIs. Furthermore, the results from the whole brain analysis show subtly different patterns of activation when contrasting reversal minus stimulus-set change and reversal minus acquisition switches. In the former case, the frontopolar spread of activation, in association with premotor and parietal cortex, accords particularly well with the pattern of activation that was observed for a similar contrast in our previous study (Hampshire and Owen, 2006) and that was shown to be under activated during reversal learning in patients with OCD (Chamberlain et al., 2008). In the latter case the activation spread is more posterior, and clearly overlaps with the pattern that is typically observed during task conditions that require the application of deliberate control. Activation within this more posterior LOFC region cannot be ascribed specifically to the processing or evaluation of negative feedback events that lead to changes in behaviour as similar coordinates have been reported when contrasting cross category and within category switches — both of which follow negative feedback (Hampshire and Owen, 2006). Nor can they be explained specifically in terms of inhibitory control, as similar coordinates have been reported during switches to previously avoided objects when there is no dominant competing response to override (Greening et al., 2011). More broadly, a similar area has often been reported in task manipulations that involve no obvious reward component at all — for example, response inhibition in the stop signal task (Aron et al., 2004; Dodds et al., 2011; Hampshire et al., 2010; Rubia et al., 2003), simple target detection (Hampshire et al., 2009; Linden et al., 1999) and the resolution of ambiguous object discriminations (Hampshire et al., 2008b). Further research is required to determine the basis of the anterior posterior LOFC dissociation observed here. However, one possible explanation, is that this difference in activation profile across the switch events results from more anterior regions being involved in the re-evaluation of contingencies (Nahum et al., 2009; O'Doherty et al., 2004), a process which is also undertaken to a lesser extent during switches in the acquisition phase. Conversely, more posterior regions may play a direct role when effortful control is applied in general (Hampshire et al., 2010), in this case to modulate the weights of competing stimulus–response mappings (Greening et al., 2011) so as to override a routine response. This type of hierarchal functional axis would be analogous to that which is believed to exist in dorsal frontal-lobe areas (Koechlin et al., 2003).

### The medial OFC and the processing of positive feedback

The sensitivity of the LOFC and LPFC to conditions that require changes in behaviour, contrasts strongly with the response that is observed in the medial OFC, a result that accords well with the mounting evidence for a functional divide between the medial and lateral OFC (Elliott et al., 2000; Kringelbach, 2005; O'Doherty et al., 2001). Specifically, the MOFC showed little if any significant response during switching; often being deactivated relative to the implicit task baseline when the participants switched to selecting a different pattern. Instead, a strong linear correlation was evident, between MOFC activation and the rated attractiveness of the faces that were used here as reinforcers. This correlation adds to the wealth of evidence supporting a role for the MOFC in the processing (Elliott et al., 2000; Hampshire and Owen, 2006; Kringelbach, 2005; Kringelbach et al., 2003; O'Doherty, 2003; O'Doherty et al., 2001, 2003, 2004) and evaluation (FitzGerald et al., 2009; Noonan et al., 2010) of rewarding, positive affective inputs.

### A role for the ACC and CN in the initiation of search behaviour?

It has previously been reported that both the ACC and the CN play a role in reversal learning and it has been suggested that the ACC and LOFC have a strong functional correlation (Kringelbach, 2005; Petrovic et al., 2002). Consistent with these accounts, in the current study both the ACC and CN ROIs were strongly activated at the point of reversal and much less so during acquisition switches. However, intriguingly, the ACC and CN, unlike the LOFC, were equally activated at the point of stimulus-set change. One prominent hypothesis, proposes that the ACC is particularly involved when there is a response conflict (Botvinick et al., 2004; Bush et al., 2000; Carter et al., 1998). An alternative hypothesis proposes that the ACC is involved more generally in representing and updating the relationship between actions and their outcomes under conditions of uncertainty, when subjects must track contingencies over multiple trials in order to guide decision making (Rushworth and Behrens, 2008). The current findings do not accord particularly well with either of these hypotheses. In the former case, ACC activation would be predicted to be particularly high during reversal relative to stimulus-set change due to the requirement to overcome the prepotent response developed over the criterion phase. In the latter case, whilst uncertainty is high at the point of stimulus-set change, activity would be predicted to be low as all stimuli available are new and consequently there are no previous trials to track. Of course, the ACC is a large structure, and quite possibly heterogeneous in function. It is possible, therefore, that the ACC ROIs examined in the current study are distinct from the rather posterior locus that has been associated with response conflict in the previous literature (Barch et al., 2001). To address this issue, we carried out a supplementary analysis on data extracted from a 5 mm spherical ROI positioned at coordinates that have previously been reported for response conflict (x = 1 y = 10 z = 46 averaged and transformed into MNI space from Barch et al., 2001). Activation was still greatest for the stimulus-set change and reversal events at this more posterior locus, although it should be noted that the results were qualitatively different from the anatomical ACC ROI with significant activation also evident for switch events in the acquisition phase. One possibility that conforms more closely with our results, is that both the ACC and the CN are particularly involved in initiating new searches, as this process is common to both reversal and stimulus-set change, but not to acquisition switches or non-switches in the contingency/criterion phase. Such a role may accord better with the recently proposed hypothesis that medial frontal-lobe areas work to innervate lateral regions (Kouneiher et al., 2009), with the ACC working to update the representation within those frontal-lobe areas that are believed to code for the overarching task schema. Similarly, based on a recent review of this literature, it has been suggested that the caudate nuclei contribute to behaviour through the excitation of action schemas and the selection of appropriate sub-goals based upon an evaluation of action outcomes (for a review see (Grahn et al., 2008)). Unlike the anatomical ACC ROIs, the CN ROIs were also significantly activated during acquisition switches, only to a lesser extent. The finding of activity in the CN in response switching is consistent with findings from lesion and electrophysiological recording studies in animals (Pasupathy and Miller, 2005; Yin et al., 2005) and imaging in humans (Arana et al., 2003; Knutson et al., 2001; Tricomi et al., 2004).

In conclusion, the present study has confirmed that different sub-regions of the reversal learning network can be dissociated when different stages of the reversal learning process are examined. It logically follows that different regions preferentially support different aspects of executive function with optimal adaptive behaviour emerging from the interactions of this cognitively heterogeneous executive network as a whole.

## Figures and Tables

**Table 1:** Results from the group level ROI analyses.

Contrast	ROI	Hemisphere	Con	t	Corrected P
a) Reversal — contingency negative	Inferior triangularis	Left	2.1	4.62	**< 0.001**
Right	1.25	2.98	**0.025**
Inferior operculum	Left	1.55	3.14	**0.016**
Right	1.5	3.62	**0.003**
Inferior orbitalis	Left	1.2	2.86	**0.036**
Right	1.2	2.68	*0.060*
Mid orbitalis	Left	1.34	4.58	**< 0.001**
Right	2.05	4.55	**< 0.001**
Med orbitalis	Left	− 0.63	− 1.27	1.000
Right	− 0.57	− 0.91	1.000
Anterior cingulate	Left	0.82	1.67	0.508
Right	1.39	2.22	0.183
Caudate nucleus	Left	1.79	3.55	**0.004**
Right	1.69	3.05	**0.020**
b) Reversal — stimulus-set change	Inferior triangularis	Left	0.06	0.17	1.000
Right	0.11	0.33	0.999
Inferior operculum	Left	− 0.46	− 1.2	1.000
Right	0.25	0.78	0.968
Inferior orbitalis	Left	0.64	1.95	0.320
Right	1.17	3.36	**0.008**
Mid orbitalis	Left	0.59	2.6	*0.073*
Right	1.47	4.18	**< 0.001**
Med orbitalis	Left	− 0.49	− 1.28	1.000
Right	− 0.96	− 1.97	1.000
Anterior cingulate	Left	− 0.02	− 0.05	1.000
Right	0.49	1.01	0.911
Caudate nucleus	Left	− 0.06	− 0.14	1.000
Right	0.07	0.15	1.000
c) Reversal — acquisition switch	Inferior triangularis	Left	0.28	0.77	0.970
Right	0.14	0.42	0.997
Inferior operculum	Left	− 0.09	− 0.23	1.000
Right	0.21	0.62	0.987
Inferior orbitalis	Left	1.45	4.2	**< 0.001**
Right	1.48	4.03	**< 0.001**
Mid orbitalis	Left	0.53	2.22	0.182
Right	0.85	2.3	0.152
Med orbitalis	Left	0.56	1.38	0.711
Right	0.9	1.75	0.447
Anterior cingulate	Left	1.33	3.32	**0.009**
Right	2.04	3.99	**< 0.001**
Caudate nucleus	Left	0.94	2.27	0.163
Right	0.97	2.13	0.221
d) Stimulus-set change — acquisition switch	Inferior triangularis	Left	0.23	0.91	0.941
Right	0.04	0.16	1.000
Inferior operculum	Left	0.36	1.34	0.739
Right	− 0.04	− 0.18	1.000
Inferior orbitalis	Left	0.81	3.49	**0.005**
Right	0.31	1.24	0.801
Mid orbitalis	Left	− 0.06	− 0.36	1.000
Right	− 0.62	− 2.49	1.000
Med orbitalis	Left	1.06	3.86	**< 0.001**
Right	1.86	5.4	**< 0.001**
Anterior cingulate	Left	1.35	5.01	**< 0.001**
Right	1.55	4.51	**< 0.001**
Caudate nucleus	Left	1	3.58	**0.004**
Right	0.9	2.96	**0.027**
e) Correlation with VAS	Inferior triangularis	Left	− 0.37	− 1.61	0.852
Right	− 1.09	− 3.6	**0.033**
Inferior operculum	Left	− 0.04	− 0.21	1.000
Right	− 1.24	− 4.05	**0.013**
Inferior orbitalis	Left	− 0.23	− 1.06	0.994
Right	− 0.77	− 2.6	0.238
Mid orbitalis	Left	0.04	0.21	1.000
Right	− 0.5	− 1.35	0.953
Med orbitalis	Left	1.06	4.01	**0.014**
Right	1.25	5.15	**0.001**
Anterior cingulate	Left	0.66	3.05	*0.101*
Right	0.73	2.08	0.543
Caudate nucleus	Left	0.15	0.61	1.000
Right	0.28	0.97	0.998

Significant effect at the corrected threshold in bold and sub-threshold trends in italics.

**Table 2:** Results from the group level whole brain analyses.

Contrast	x	y	z	t	Region	
a) Reversal — contingency negative	− 36	48	6	5.92	Left	MFG/frontopolar cortex
− 36	33	36	5.73	Left	MFG/mid dorsolateral PFC
36	12	54	5.61	Right	MFG/premotor cortex
27	0	15	5.31	Right	Striatum
0	− 87	− 6	5.24	Medial	Occipital cortex
− 39	21	− 3	5.13	Left	Insula/operculum/posterior LOFC
9	27	36	5.01	Medial	ACC/preSMA
57	− 54	27	4.81	Right	Parietal cortex
− 42	6	42	4.63	Left	MFG/premotor cortex
27	60	− 9	4.61	Right	Anterior LOFC
− 60	− 51	30	4.54	Left	Parietal cortex
− 33	54	− 9	4.49	Left	Anterior LOFC
− 39	18	− 9	4.34	left	Insula/posterior LOFC
36	18	− 6	4.31	Right	Insula/posterior LOFC
18	12	− 9	4.31	Right	Striatum
− 48	21	− 6	4.12	Left	Posterior LOFC
39	57	6	4.06	Right	MFG/frontopolar cortex
57	15	0	4.04	Right	Insula/operculum
− 27	6	6	3.86	Left	Striatum
48	33	27	3.85	Right	MFG/inferior triangularis
51	18	− 9	3.67	Right	Posterior LOFC
− 15	9	− 12	3.64	Left	Striatum
b) Reversal — stimulus-set change	36	48	− 3	5.99	Right	Anterior LOFC
54	33	− 12	5.96	Right	Posteror LOFC
6	39	51	5.27	Medial	Superior frontal gyrus
− 30	21	− 9	4.95	Left	Posteror LOFC/insula
36	21	− 9	4.61	Right	Posteror LOFC/insula
45	18	45	4.46	Right	MFG/premotor cortex
− 45	48	− 12	3.74	Left	Anterior LOFC
48	− 51	33	3.68	Right	Parietal cortex
36	48	18	3.38	Right	MFG/premotor cortex
c) Reversal — acquisition switch	− 27	21	− 12	6.23	Left	Posterior LOFC/insula
57	27	− 9	6.17	Right	Posterior LOFC
3	− 18	36	5.33	Medial	Posterior cingulate
− 9	3	− 12	5.1	Left	Striatum
36	21	− 12	5.07	Right	Posterior LOFC/insula
0	− 48	30	4.95	Medial	Posterior cingulate
− 39	36	− 12	4.92	Left	Posterior LOFC
6	39	54	4.73	Medial	Superior frontal gyrus
3	30	30	4.63	Medial	ACC/preSMA
54	− 21	− 12	4.33	Right	Middle temporal gyrus
6	48	21	4.32	Medial	ACC/preSMA
12	12	6	4.21	Right	Striatum
27	9	− 9	4.12	Right	Striatum
− 6	6	3	4.09	Left	Striatum
36	48	− 3	3.61	Right	Anterior LOFC
− 51	42	− 9	3.46	Left	Anterior LOFC
d) Stimulus-set change — acquisition switch	− 21	− 18	− 12	7.01	Left	Medial temporal lobe
30	− 18	− 15	6.48	Right	Medial temporal lobe
− 48	− 9	− 6	6.02	Left	Middle temporal gyrus
0	39	6	5.66	Medial	ACC
3	45	− 6	5.56	Medial	MOFC
3	− 30	39	5.55	Medial	Posterior cingulate
24	6	− 9	5.16	Right	Striatum
48	− 15	− 12	5.06	Right	Middle temporal gyrus
− 15	12	− 3	5.03	Left	Striatum
e) VAS positive	− 6	− 57	18	7.37	Medial	Precueneus
0	48	− 3	6.82	Medial	ACC/MOFC
51	− 30	27	6.76	Right	Temporal parietal junction
0	36	− 6	6.47	Medial	ACC/MOFC
− 27	− 36	− 12	5.8	Left	Medial temporal lobe
− 57	− 30	36	5.56	Left	Temporal parietal junction
f) VAS negative	36	24	3	10.75	Right	Insula/operculum/inferior triangularis
6	18	48	8.1	Medial	Supplementary motor area
− 27	45	21	7.4	Left	MFG/mid DLPFC
− 39	18	0	6.65	Left	Insula/operculum

## References

[bb0005] Aharon I., Etcoff N., Ariely D., Chabris C.F., O'Connor E., Breiter H.C. (2001). Beautiful faces have variable reward value: fMRI and behavioral evidence. Neuron.

[bb0010] Arana F.S., Parkinson J.A., Hinton E., Holland A.J., Owen A.M., Roberts A.C. (2003). Dissociable contributions of the human amygdala and orbitofrontal cortex to incentive motivation and goal selection. J. Neurosci..

[bb0015] Aron A.R., Robbins T.W., Poldrack R.A. (2004). Inhibition and the right inferior frontal cortex. Trends Cogn. Sci..

[bb0020] Barch D.M., Braver T.S., Akbudak E., Conturo T., Ollinger J., Snyder A. (2001). Anterior cingulate cortex and response conflict: effects of response modality and processing domain. Cereb. Cortex.

[bb0025] Botvinick M.M., Cohen J.D., Carter C.S. (2004). Conflict monitoring and anterior cingulate cortex: an update. Trends Cogn. Sci..

[bb0030] Brett M., Anton J., Valabregue R., Poline J. (2002). Region of interest analysis using an SPM toolbox [abstract]. 8th International Conference on Functional Mapping of the Human Brain, Sendai, Japan.

[bb0035] Budhani S., Marsh A.A., Pine D.S., Blair R.J. (2007). Neural correlates of response reversal: considering acquisition. NeuroImage.

[bb0040] Bush G., Luu P., Posner M.I. (2000). Cognitive and emotional influences in anterior cingulate cortex. Trends Cogn. Sci..

[bb0045] Carter C.S., Braver T.S., Barch D.M., Botvinick M.M., Noll D., Cohen J.D. (1998). Anterior cingulate cortex, error detection, and the online monitoring of performance. Science.

[bb0050] Chamberlain S.R., Menzies L., Hampshire A., Suckling J., Fineberg N.A., del Campo N., Aitken M., Craig K., Owen A.M., Bullmore E.T., Robbins T.W., Sahakian B.J. (2008). Orbitofrontal dysfunction in patients with obsessive–compulsive disorder and their unaffected relatives. Science.

[bb0055] Chudasama Y., Robbins T.W. (2003). Dissociable contributions of the orbitofrontal and infralimbic cortex to pavlovian autoshaping and discrimination reversal learning: further evidence for the functional heterogeneity of the rodent frontal cortex. J. Neurosci..

[bb0295] Clarke H.F., Roberts A.C., Delgado M.R., Phelps E.A., Robbins T.W. (2011). Reversal learning in fronto-striatal circuits: a functional, autonomic and neurochemical analysis. Attention and Performance XXIII, pp.

[bb0060] Clarke H.F., Robbins T.W., Roberts A.C. (2008). Lesions of the medial striatum in monkeys produce perseverative impairments during reversal learning similar to those produced by lesions of the orbitofrontal cortex. J. Neurosci..

[bb0065] Cools R., Clark L., Owen A.M., Robbins T.W. (2002). Defining the neural mechanisms of probabilistic reversal learning using event-related functional magnetic resonance imaging. J. Neurosci..

[bb0070] Cusack R., Brett M., Osswald K. (2003). An evaluation of the use of magnetic field maps to undistort echo-planar images. NeuroImage.

[bb0075] Dias R., Robbins T.W., Roberts A.C. (1996). Dissociation in prefrontal cortex of affective and attentional shifts. Nature.

[bb0080] Dias R., Robbins T.W., Roberts A.C. (1997). Dissociable forms of inhibitory control within prefrontal cortex with an analog of the Wisconsin Card Sort Test: restriction to novel situations and independence from “on-line” processing. J. Neurosci..

[bb0085] Dodds C.M., Morein-Zamir S., Robbins T.W. (2011). Dissociating inhibition, attention, and response control in the frontoparietal network using functional magnetic resonance imaging. Cereb. Cortex.

[bb0090] Elliott R., Dolan R.J., Frith C.D. (2000). Dissociable functions in the medial and lateral orbitofrontal cortex: evidence from human neuroimaging studies. Cereb. Cortex.

[bb0095] Fellows L.K., Farah M.J. (2003). Ventromedial frontal cortex mediates affective shifting in humans: evidence from a reversal learning paradigm. Brain.

[bb0100] FitzGerald T.H., Seymour B., Dolan R.J. (2009). The role of human orbitofrontal cortex in value comparison for incommensurable objects. J. Neurosci..

[bb0105] Ghahremani D.G., Monterosso J., Jentsch J.D., Bilder R.M., Poldrack R.A. (2010). Neural components underlying behavioral flexibility in human reversal learning. Cereb. Cortex.

[bb0110] Grahn J.A., Parkinson J.A., Owen A.M. (2008). The cognitive functions of the caudate nucleus. Prog. Neurobiol..

[bb0115] Greening S.G., Finger E.C., Mitchell D.G. (2011). Parsing decision making processes in prefrontal cortex: response inhibition, overcoming learned avoidance, and reversal learning. NeuroImage.

[bb0120] Hampshire A., Owen A.M. (2006). Fractionating attentional control using event-related fMRI. Cereb. Cortex.

[bb0125] Hampshire A., Gruszka A., Fallon S.J., Owen A.M. (2008). Inefficiency in self-organized attentional switching in the normal aging population is associated with decreased activity in the ventrolateral prefrontal cortex. J. Cogn. Neurosci..

[bb0130] Hampshire A., Thompson R., Duncan J., Owen A.M. (2008). The target selective neural response—similarity, ambiguity, and learning effects. PLoS One.

[bb0135] Hampshire A., Thompson R., Duncan J., Owen A.M. (2009). Selective tuning of the right inferior frontal gyrus during target detection. Cogn. Affect. Behav. Neurosci..

[bb0140] Hampshire A., Chamberlain S.R., Monti M.M., Duncan J., Owen A.M. (2010). The role of the right inferior frontal gyrus: inhibition and attentional control. NeuroImage.

[bb0145] Hornak J., O'Doherty J., Bramham J., Rolls E.T., Morris R.G., Bullock P.R., Polkey C.E. (2004). Reward-related reversal learning after surgical excisions in orbito-frontal or dorsolateral prefrontal cortex in humans. J. Cogn. Neurosci..

[bb0150] Iversen S.D., Mishkin M. (1970). Perseverative interference in monkeys following selective lesions of inferior prefrontal convexity. Exp. Brain Res..

[bb0155] Izquierdo A., Suda R.K., Murray E.A. (2004). Bilateral orbital prefrontal cortex lesions in rhesus monkeys disrupt choices guided by both reward value and reward contingency. J. Neurosci..

[bb0160] Knutson B., Adams C.M., Fong G.W., Hommer D. (2001). Anticipation of increasing monetary reward selectively recruits nucleus accumbens. J. Neurosci..

[bb0165] Koechlin E., Ody C., Kouneiher F. (2003). The architecture of cognitive control in the human prefrontal cortex. Science.

[bb0170] Kouneiher F., Charron S., Koechlin E. (2009). Motivation and cognitive control in the human prefrontal cortex. Nat. Neurosci..

[bb0175] Kringelbach M.L. (2005). The human orbitofrontal cortex: linking reward to hedonic experience. Nat. Rev. Neurosci..

[bb0180] Kringelbach M.L., O'Doherty J., Rolls E.T., Andrews C. (2003). Activation of the human orbitofrontal cortex to a liquid food stimulus is correlated with its subjective pleasantness. Cereb. Cortex.

[bb0185] Linden D.E.J., Prvulovic D., Formisano E., Vollinger M., Zanella F.E., Goebel R., Dierks T. (1999). The functional neuroanatomy of target detection: An fMRI study of visual and auditory oddball tasks. Cereb. Cortex.

[bb0190] Liu X., Powell D.K., Wang H.B., Gold B.T., Corbly C.R., Joseph J.E. (2007). Functional dissociation in frontal and striatal areas for processing of positive and negative reward information. J. Neurosci..

[bb0195] Mitchell D.C., Bryan B.A. (2010). Anti-angiogenic therapy: adapting strategies to overcome resistant tumors. J. Cell. Biochem..

[bb0200] Nahum L., Ptak R., Leemann B., Schnider A. (2009). Disorientation, confabulation, and extinction capacity: clues on how the brain creates reality. Biol. Psychiatry.

[bb0205] Noonan M.P., Walton M.E., Behrens T.E., Sallet J., Buckley M.J., Rushworth M.F. (2010). Separate value comparison and learning mechanisms in macaque medial and lateral orbitofrontal cortex. Proc. Natl. Acad. Sci. U. S. A..

[bb0210] O'Doherty J. (2003). Can't learn without you: predictive value coding in orbitofrontal cortex requires the basolateral amygdala. Neuron.

[bb0215] O'Doherty J., Kringelbach M.L., Rolls E.T., Hornak J., Andrews C. (2001). Abstract reward and punishment representations in the human orbitofrontal cortex. Nat. Neurosci..

[bb0220] O'Doherty J., Winston J., Critchley H., Perrett D., Burt D.M., Dolan R.J. (2003). Beauty in a smile: the role of medial orbitofrontal cortex in facial attractiveness. Neuropsychologia.

[bb0225] O'Doherty J., Dayan P., Schultz J., Deichmann R., Friston K., Dolan R.J. (2004). Dissociable roles of ventral and dorsal striatum in instrumental conditioning. Science.

[bb0230] Pasupathy A., Miller E.K. (2005). Different time courses of learning-related activity in the prefrontal cortex and striatum. Nature.

[bb0235] Petrovic P., Kalso E., Petersson K.M., Ingvar M. (2002). Placebo and opioid analgesia—imaging a shared neuronal network. Science.

[bb0240] Rogers R.D., Andrews T.C., Grasby P.M., Brooks D.J., Robbins T.W. (2000). Contrasting cortical and subcortical activations produced by attentional-set shifting and reversal learning in humans. J. Cogn. Neurosci..

[bb0245] Rubia K., Smith A.B., Brammer M.J., Taylor E. (2003). Right inferior prefrontal cortex mediates response inhibition while mesial prefrontal cortex is responsible for error detection. NeuroImage.

[bb0250] Rushworth M.F., Behrens T.E. (2008). Choice, uncertainty and value in prefrontal and cingulate cortex. Nat. Neurosci..

[bb0255] Rygula R., Walker S.C., Clarke H.F., Robbins T.W., Roberts A.C. (2010). Differential contributions of the primate ventrolateral prefrontal and orbitofrontal cortex to serial reversal learning. J. Neurosci..

[bb0260] Schnider A. (2003). Spontaneous confabulation and the adaptation of thought to ongoing reality. Nat. Rev. Neurosci..

[bb0265] Schoenbaum G., Nugent S.L., Saddoris M.P., Setlow B. (2002). Orbitofrontal lesions in rats impair reversal but not acquisition of go, no-go odor discriminations. NeuroReport.

[bb0270] Solina F., Peer P., Batagelj B., Juvan S., Kovac J., Philips W. (2003). Color-based face detection in the “15 seconds of fame” art installation. Conference on Computer Vision/ Computer Graphics Collaboration for Model-based Imaging, Rendering, Image Analysis and Graphical special Effects.

[bb0275] Tricomi E.M., Delgado M.R., Fiez J.A. (2004). Modulation of caudate activity by action contingency. Neuron.

[bb0280] Tzourio-Mazoyer N., Landeau B., Papathanassiou D., Crivello F., Etard O., Delcroix N. (2002). Automated anatomical labelling of activations in SPM using a macroscopic anatomical parcellation of the MNI MRI single subject brain. NeuroImage.

[bb0285] Windmann S., Kirsch P., Mier D., Stark R., Walter B., Gunturkun O., Vaitl D. (2006). On framing effects in decision making: linking lateral versus medial orbitofrontal cortex activation to choice outcome processing. J. Cogn. Neurosci..

[bb0290] Yin H.H., Ostlund S.B., Knowlton B.J., Balleine B.W. (2005). The role of the dorsomedial striatum in instrumental conditioning. Eur. J. Neurosci..

